# Inhibitory Effect of b-AP15 on the 20S Proteasome

**DOI:** 10.3390/biom4040931

**Published:** 2014-10-14

**Authors:** Li Huang, Katherine Jung, Chin Ho Chen

**Affiliations:** Department of Surgery, Duke University Medical Center, Durham, NC 27710, USA; E-Mails: li.huang@duke.edu (L.H.); kathy.jung@duke.edu (K.J.)

**Keywords:** proteasome inhibitor, b-AP15, 19S regulatory particles

## Abstract

The 26S proteasome is a cellular proteolytic complex containing 19S regulatory particles and the 20S core proteasome. It was reported that the small molecule b-AP15 targets the proteasome by inhibiting deubiquitination of the 19S regulatory particles of the proteasome complex. An investigation of b-AP15 on the 20S proteasome core suggested that this compound can also inhibit the 20S proteasome with a potency equivalent to that found to inhibit the 19S regulatory particles.

## 1. Introduction

The ubiquitin-proteasome system is important in maintaining cell homeostasis by carrying out protein degradation inside the cell [1,2]. Unwanted and damaged proteins are polyubiquitinated by an ATP-dependent process and shuttled to the proteasome complex for degradation [1,2]. The ubiquitin-proteasome pathway is regulated by a host of enzymes and regulatory proteins, many of which have been investigated as potential targets for drug intervention. A variety of important clinical problems, such as cancers, Alzheimer’s disease, inflammatory and autoimmune diseases, have been associated with dysfunction of the proteasome and thus could benefit from drugs that regulate the ubiquitin-proteasome pathway [3,4,5,6].

The 26S proteasome is a proteolytic complex containing 19S regulatory particles and the 20S core proteasome. The 20S core contains catalytic sites for proteolysis of protein/peptide substrates, while the 19S particles activate the proteasome complex and allow the substrates to access the 20S proteasome core. A considerable number of proteasome inhibitors have been discovered or synthesized with high affinity to the catalytic sites of the 20S proteasome [7,8,9]. A few of them were found to have promising therapeutic potential. For example, bortezomib and carfilzomib are potent 20S proteasome inhibitors approved in the U.S. for treating cancers, such as multiple myeloma [10,11]. On the other hand, b-AP15, a nitrophenylpiperidine small molecule, was reported to target the 19S regulatory particles of the 26S proteasome. It was indicated that b-AP15 inhibited the deubiquitinating activity of two 19S regulatory particle-associated deubiquitinases, ubiquitin C-terminal hydrolase 5 (UCHL5) and ubiquitin-specific peptidase 14 (USP14) [12]. Recently, b-AP15 was shown to have anti-multiple myeloma activity in a human xenograft mouse model [13]. There is considerable interest in b-AP15 as a drug candidate due to its distinct mode of action in comparison to other known proteasome inhibitors, such as bortezomib that targets the 20S proteasome core particle.

## 2. Results and Discussion

### 2.1. b-AP15 Potently Inhibited the Proteasome in a Cell-Based Assay

**Figure 1:**
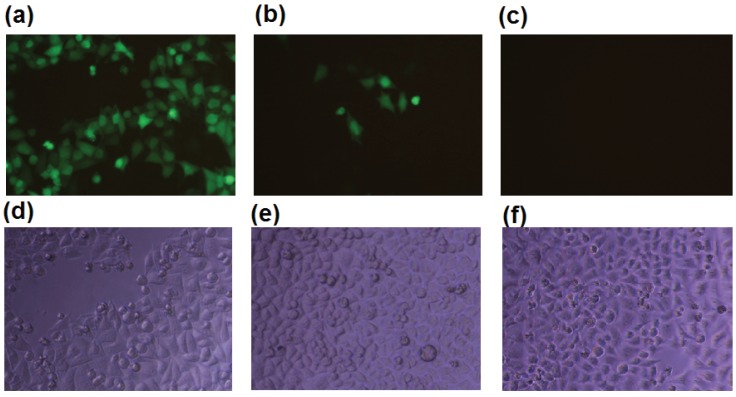
b-AP15 inhibited the proteasome of HeLa ubiquitin-green fluorescent protein (Ub^G76V^-GFP) cells. HeLa Ub^G76V^-GFP cells were treated with b-AP15 at 0.95 μM (**a,d**); 0.32 μM (**b,e**), or no b-AP15 (**c,f**) for 24 h, where (**a**), (**b**), and (**c**) are fluorescence microscopic images; (**d**), (**e**), and (**f**) are regular phase contrast images of the cells.

It was shown that b-AP15 at 50 μM inhibited the deubiquitination by the 19S regulatory particle, but not the 20S proteasome in *in vitro* enzymatic assays [12]. In the process of studying the effect of b-AP15 on the proteasome in intracellular milieu, we found that the compound strongly inhibited the proteasome in HeLa Ub^G76V^-GFP cells at sub-micromolar concentrations (Figure 1). HeLa Ub^G76V^-GFP cells express ubiquitin-green fluorescent protein (Ub-GFP), which is efficiently degraded by the proteasome [14]. HeLa Ub^G76V^-GFP cells accumulate Ub-GFP in the presence of proteasome inhibitors. Treatment of the HeLa Ub^G76V^-GFP cells with 0.95 μM of b-AP15 resulted in accumulation of *Ub^G76V^-GFP and/or GFP* in majority of the cells (Figure 1a). Some of the HeLa Ub^G76V^-GFP cells turned green in the presence of b-AP15 at a concentration as low as 0.32 μM (Figure 1b). The potency of b-AP15 determined with the HeLa Ub^G76V^-GFP cell model was comparable to that when a MelJuSo Ub-YFP reporter cell line was used in a similar assay [12]. Ub-YFP was accumulated in the MelJuSo Ub-YFP cells at a b-AP15 concentration as low as 0.7 μM [12].

### 2.2. b-AP15 Inhibited Both 19S Regulatory Particle and the 20S Proteasome

**Figure 2:**
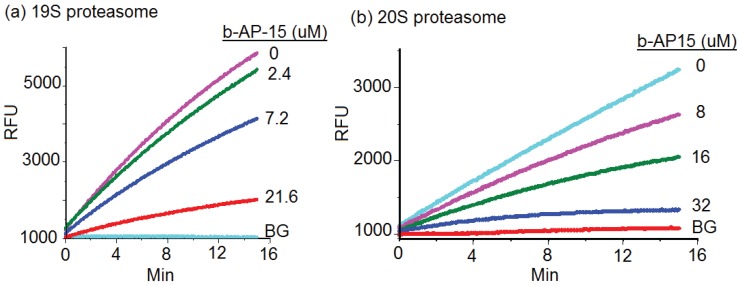
(**a**) b-AP15 inhibited the 19S regulatory particles. The activity of human 19S regulatory particles (Boston Biochem) was determined in the presence of the fluorogenic substrate ub-AMC and various concentrations of b-AP15 as indicated using a reported assay condition [12]. RFU denotes the relative fluorescence units of the reactions; (**b**) Inhibitory effect of b-AP15 on the chymotrypsin-like activity of the 20S proteasome. BG in the figure denotes background.

By disabling the function of 19S particle, b-AP15 was expected to inhibit the degradation of Ub^G76V^-GFP. However, b-AP15 exhibited relatively weak inhibitory activity in 19S regulatory particle enzymatic assays. As shown in Figure 2a, b-AP15 inhibited the deubiquitinase activities of the 19S regulatory particles with an IC_50_ of 15.2 μM. The slope of each line in the figure represents the reaction rate of deubiquitination activities of the 19S regulatory particles.

Wang *et al*. recently reported that there was a thiol-dependent enrichment of b-AP15 in cells [15]. This may be responsible for the relatively potent intracellular activity of b-AP15 displayed in the HeLa Ub^G76V^-GFP cell assay (Figure 1). However, the intracellular free b-AP15 concentration was rather low (1–5 nM) [15]. This raised the possibility that b-AP15 might have targeted the proteasome at multiple sites to exert such potent inhibitory activity. To test this possibility, the effect of b-AP15 on the 20S proteasome core particle was determined using a standard in vitro assay for the chymotrypsin-like activity of the 20S proteasome [16,17]. An example of inhibition of the chymotrypsin-like activity of the 20S proteasome by b-AP15 was shown in Figure 2b. b-AP15 inhibited the chymotrypsin-like activity of the 20S proteasome with a 50% inhibitory concentration (IC_50_) at 18.1 μM (Table 1), a potency comparable to that of 19S regulatory particle inhibition (15.2 μM).

The 20S proteasome assay used in this experiment involved activation of the 20S proteasome by the proteasome activator PA28. PA28 is a cellular proteasome activator commonly used to activate the 20S proteasome in enzymatic assays. b-AP15 could have inhibited the proteasomal activity by targeting either the 20S core particle or PA28. In order to determine whether the 20S proteasome was a target of b-AP15, we used 3α-*N*-pimeloyl-amino-lithocholic acid methyl ester (PALAME), a small molecule proteasome activator, to activate the 20S proteasome [16]. b-AP15 inhibited the PALAME-activated chymotrypsin-like activity at concentrations comparable to that when PA28 was used as an activator. The compound inhibited the PALAME-activated 20S proteasome with an IC_50_ of 26 μM (Table 1). This result suggests that the 20S proteasome is a target of b-AP15.

**Table 1:** Inhibitory effect of b-AP15 on the 20S proteasome *^a^*.

		IC_50_ (μM)	
Compound	PA28	PALAME	SDS
b-AP15	18.1 ± 3.22	26.3 ± 3.15	>48
	>48 *^b^*		
	40.6 ± 5.23 *^c^*		
Lactacystin	5.8 ± 0.76	5.3 ± 0.68	6.4 ± 0.71
*Bortezomib*	0.0078 ±0.0012	0.0065 ±0.00093	0.0087 ±0.00015

*^a^* The effects of b-AP15 or lactacystin on the chymotrypsin-like activity of the 20S proteasome activated by PA28, PALAME, or SDS; *^b,c^* The effects of b-AP15 on the caspase-like *^b^* and the trypsin-like *^c^* activities of PA28-activated 20S proteasome, respectively.

It was reported by D’Arcy *et al.* that b-AP15 did not inhibit the 20S proteasome [12]. A major difference between the assay described here and that described by D’Arcy *et al.* lies within the reagents used for proteasome activation. SDS was used by D’Arcy *et al.* to activate the 20S proteasome, whereas PA28 or PALAME was used to activate the proteasome in this study. Thus, the effect of b-AP15 on the 20S proteasome was also determined using 0.03% SDS as an activator. b-AP15 at 48 μM inhibited less than 50% of the chymotrypsin-like activity of the SDS-activated 20S proteasome (Table 1). On the other hand, the known active site proteasome inhibitor, lactacystin, inhibited the SDS-activated proteasome at concentrations comparable to that of PA28 or PALAME-activated 20S proteasome (Table 1). SDS is thought to activate the 20S proteasome by partially denaturing the proteasome, which allows substrates to access catalytic sites in the 20S proteasome. In contrast, PA28 and PALAME are believed to activate the proteasome by opening the gate through induction of conformational changes [16,18]. b-AP15 may be an allosteric inhibitor that is inefficient in blocking the chaotropic effect of SDS on the proteasome, but capable of arresting the conformational changes induced by PA28 or PALAME.

**Figure 3:**
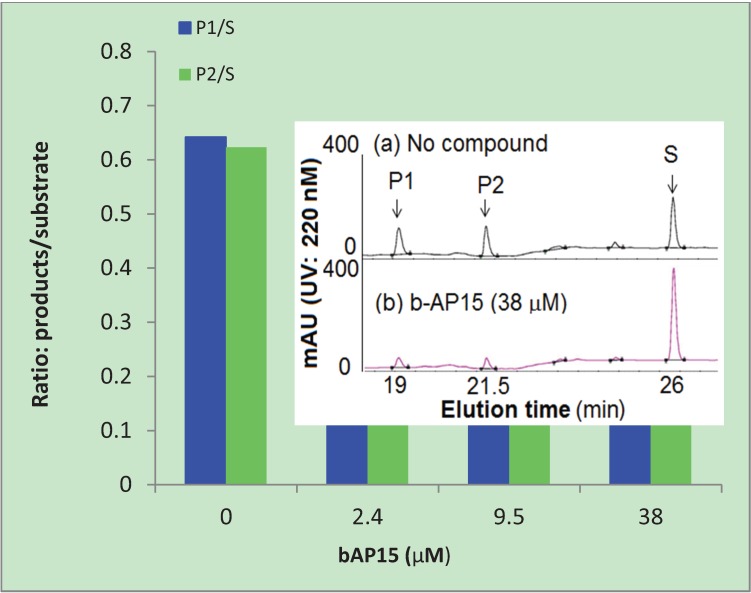
Inhibition of the chymotrypsin-like activity of the 20S proteasome. The reaction products of the 20S proteasome, AMC (P1), Suc-LLVY (P2), and substrate Suc-LLVY-AMC (S) were analyzed by HPLC. P1/S and P2/S are the ratios of the reaction products and the substrate calculated from the area under the curve of the HPLC profiles.

There are three major proteolysis activities in the proteasome: chymotrypsin-like, trypsin-like, and caspase-like activities. To test whether b-AP15 also inhibited the trypsin-like and caspase-like activities, the 20S proteasome was assayed in the presence of various concentration of b-AP15 using the same assay protocol previously described [16,17]. b-AP15 did not significantly affect the caspase-like activity at a concentration as high as 48 μM (Table 1). On the other hand, b-AP15 weakly inhibited the trypsin-like activity with an IC_50_ of 40.6 μM. These results suggest that b-AP15 is able to inhibit two of the three major proteolytic activities of the 20S proteasome. The differential sensitivity might be due to different binding kinetics of b-AP15 to the β5, β2, and β1 subunits of the proteasome. The chymotrypsin-like, trypsin-like, and caspase-like activities are located in the β5, β2, and β1 subunits of the proteasome, respectively [19]. On the other hand, the lack of inhibitory activity against the PA28-activated caspase-like activity suggests that b-AP15 did not significantly affect binding of PA28 to the 20S proteasome. Although b-AP15 did not inhibit the PA28-activated caspase-like activity, the effect of the compound on the 19S regulatory particle-activated caspase-like activity of the 20S proteasome, or the association between the 19S and 20S proteasome, was not clear.

b-AP15 is highly chromogenic with a conjugated π-bonding system that could potentially be quenching the emitted fluorescence from proteasome cleavage of the substrate. Diminished fluorescence by means of quenching could affect the interpretation of the results shown in Table 1. To eliminate the possible effect of quenching by b-AP15, we used HPLC to analyze the substrate and cleaved products, AMC (P1) and Suc-LLVY (P2), after the enzyme reactions. The ratio of P/S was used to express the relative amount of reaction product formation under the assay conditions, where P represents the products and S represents the substrate Suc-LLVY-AMC (S). As shown in Figure 3, b-AP15 inhibited the formation of the two cleaved products in a dose dependent manner. The inset in the figure is a representative HPLC profile of the reaction mixtures that contain the substrate Suc-LLVY-AMC and the two main cleaved products, P1 and P2. It was clear that the two main cleaved products markedly diminished in the presence of b-AP15.

## 3. Experimental

### 3.1. Cell-Based Proteasome Inhibition Assay

HeLa Ub^G76V^-GFP cells (kindly provided by Nico P. Dantuma, Karolinska Institutet, Stockholm, Sweden) [14] were cultured in DMEM medium supplemented with 10% fetal calf serum, 100 units/mL of penicillin, and 100 µg/mL of streptomycin. The cells were seeded in a 96-well plate at 5 × 10^3^ per well, a day before adding proteasome inhibitors. The cells were treated with b-AP15 (Calbiochem/EMD Millipore and, in part, kindly provided by Stig Linder, Karolinska Institute, City, Sweden) at various concentrations for 24 h. The green fluorescence emitted by the treated HeLa Ub^G76V^-GFP cells was then observed and documented with a Nikon fluorescent microscopic system.

### 3.2. 19S Regulatory Particle De-Ubiquitinase Assay

The de-ubiquitinase activities of the human 19S regulatory particles (proteasome) (Boston Biochem, Boston, MA, USA) were determined in the presence of the fluorogenic substrate ub-AMC (Boston Biochem) and various concentrations of b-AP15 using the reported assay condition [12]. The reaction buffer used contained 25 mM HEPES, 50 mM NaCl, 10 mM MgCl_2_, 2 mM ATP, and 1 mM DDT. The human 19S regulatory particles and the substrate ub-AMC were used at a final concentration of 2.9 nM, and 1 μM, respectively. The proteolysis reaction rate was monitored using a BioTek fluorometer (Winooski, VT, USA).

### 3.3. 20S Proteasome Assay

Proteasome assay kits were purchased from Calbiochem, San Diego, CA. The effect of the proteasome inhibitors on the 20S proteasome activities was assayed following the protocols provided by the manufacturer. The major components of the assay mixture are the human 20S proteasome, fluorogenic peptide substrates and a proteasome activator. The chymotrypsin-like activity of the 20S proteasome was assayed by incubating the proteasome (0.5 μg/mL) with the ﬂuorogenic substrate Suc-LLVY-AMC (20 μM) in the presence of a proteasome activator and b-AP15 or lactacystin. The concentrations of the proteasome activators used, PA28, PALAME, and SDS were 2 μg/mL, 19 μM, and 0.03% (V/V), respectively. PA28, also known as 11S, has been shown to activate the 20S proteasomes against model peptide substrates [20,21].

The trypsin-like and caspase-like activities of the 20S proteasome were determined using the fluorogenic substrates Bz-VGR-AMC (20 μM) and (Z)-LLEbNA (20 μM), respectively. Fluorescence generated from the proteolytic reaction in the presence of various concentrations of the inhibitors was measured using a BioTek fluorometer. The known proteasome inhibitor lactacystin was used as a control for the proteasome inhibition assays. The 50% inhibitory concentration (IC_50_) is defined as the inhibitory concentration that reduces the reaction rate by 50%. The value of IC_50_ is expressed as mean ± standard deviation from three independent assays using CalcuSyn (Biosoft, Cambridge, UK).

### 3.4. HPLC Analysis of the Substrates and Products of the 20S Proteasome

b-AP15 at various concentrations (0, 2.4, 9.6, and 38 μM) was incubated with the 20S proteasome (4.3 nM), substrate (Suc-LLVY-AMC, 0.2 mM), and PA28 (11 μg/mL) at room temperature for 6 h. The reaction was terminated by filtration with Amicon Ultra Centrifugal filters (Millipore) to remove the 20S proteasome and PA28. The reaction products, AMC (P1), Suc-LLVY (P2), and substrate Suc-LLVY-AMC (S) in the filtrate were analyzed using reverse phase HPLC with a gradient of increasing acetonitrile/water ratio. HPLC was performed on a Varian ProStar solvent delivery system and a PDA detector with an Agilent Zorbax sb-C18 column (5 μm, 4.6 × 250 mm). The gradient elution had a flow rate of 1.0 mL/min. The initial elution condition was 100% of solvent A (95% water in acetonitrile with 0.045% trifluoroacetic acid). After staying at the initial condition for 3 min, the concentration of solvent B (acetonitrile/methanol/water = 85:10:5 with 0.045% trifluoroacetic acid) increased linearly from 0 to 20% at 10 min, and increased to 100% at 30 min then stayed at that level for 5 min. The UV absorption was displayed at 220 nm and recorded at a range from 200 to 380 nm. The relative quantity of the reaction products and the substrate was calculated from the area under the curve of the HPLC profiles using Galaxie chromatography software (Varian, Inc., Santa Clara, CA, USA).

## 4. Conclusions

**Figure 4:**
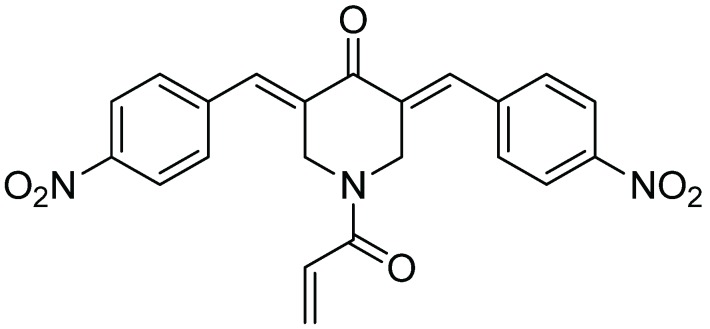
Chemical structure of b-AP15.

In summary, the data indicate that b-AP15 can inhibit the 20S proteasome with an IC_50_ comparable to that required for inhibiting the 19S regulatory particles. Thus, in addition to UCHL5 and USP14 of the 19S regulatory particle, the 20S proteasome is a relevant target for b-AP15. Targeting multiple sites on the 19S regulatory particle and the 20S proteasome might be responsible for the potent proteasome inhibitory activity in the Ub-GFP cell-based assays. The promiscuity of b-AP15 binding to multiple targets might be attributed to its chemical properties. As shown in Figure 4, the structure of b-AP15 contains multiple Michael acceptors which are capable of interacting with sulfhydryl or hydroxyl groups of amino acids in proteins. The Michael acceptor reactivity of b-AP15 and its binding to thiols were recently discussed by Wang, *et al.* [21]. This reactivity is likely responsible for the ability of b-AP15 to interact with multiple proteins.
